# Potassium Sodium Hydrogen Citrate in Managing Surgical Candidates With Urinary Stones: A Case Series

**DOI:** 10.7759/cureus.78926

**Published:** 2025-02-12

**Authors:** Yahya Ghazwani, Nasser Albogami, Fahad Barayan, Abdullah Alsaghyir, Meshari Alshaashaa, Ghassan Alhajress

**Affiliations:** 1 College of Medicine, King Saud bin Abdulaziz University for Health Sciences, Riyadh, SAU; 2 Division of Urology, Department of Surgery, Ministry of National Guard - Health Affairs, Riyadh, SAU; 3 Department of Medicine, King Abdullah International Medical Research Center, Riyadh, SAU

**Keywords:** alkaline citrate, calcium oxalate/phosphate, kidney stones, nephrolithiasis, percutaneous nephrolithotomy, saudi arabia, sodium potassium hydrogen citrate, ureteroscopy

## Abstract

Kidney stone disease is highly prevalent in Saudi Arabia, where conventional treatments often involve invasive procedures that carry significant risks. This case series examines the efficacy of potassium sodium hydrogen citrate as a non-invasive alternative for managing kidney stones. Five patients with complex medical histories, large stone sizes, and diverse stone compositions were treated with this therapy, resulting in complete stone dissolution without the need for surgical intervention. The findings highlight the potential of this approach to effectively dissolve stones and prevent recurrence, particularly for patients deemed unsuitable for surgery. Despite its proven efficacy, the adoption of chemical management remains underutilized in Saudi Arabia. Incorporating potassium sodium hydrogen citrate into the standard treatment protocols could reduce dependence on invasive procedures, improve patient outcomes, and alleviate the burden on healthcare resources.

## Introduction

Kidney stones, also known as nephrolithiasis or urolithiasis, are a common health concern that impact millions of people globally, including those residing in Saudi Arabia [1,2]. The incidence of kidney stones has been steadily increasing over the past four decades, with prevalence rates ranging from 1%-5% in Asia, 5%-9% in Europe, and 7%-13% in the USA [3]. Although comprehensive national data on urolithiasis in Saudi Arabia is lacking, estimates suggest an increase in prevalence [4], particularly in cities like Jeddah and Riyadh, where rates have reached up to 16.4% [5]. Similar trends have been reported in neighboring countries, including the United Arab Emirates and Kuwait [6]. The recurrence rates of kidney stones are also alarmingly high, with 50% of patients experiencing a recurrence in 5-10 years and 75% within 20 years [7].

Kidney stones are primarily composed of calcium oxalate, uric acid, and calcium phosphate. These minerals and salt deposits can cause severe complications such as pain, hematuria, urinary tract infections, chronic kidney diseases (CKDs), cardiovascular diseases, myocardial infarction, and even end-stage renal failure [8,9]. These complications significantly impact the patient's health-related quality of life, adding an extra economic burden for its management [10]. In Saudi Arabia, factors like hot climate, family history of kidney stone disease, primary hyperparathyroidism, dietary factors, low fluid intake, and hypercalciuria significantly contribute to the development of kidney stones [2].

Conventional treatments often involve surgical interventions such as percutaneous nephrolithotomy (PCNL), extracorporeal shock wave lithotripsy, and ureteroscopy. Although these methods are effective, they tend to be costly and pose potential risks of complications [11]. The recurrence of kidney stones remains a significant challenge, with patients frequently experiencing residual stones post-surgery. Potassium sodium hydrogen citrate has been recognized for its potential in facilitating the dissolution of kidney stones without the need for surgery and achieving optimal urinary pH in patients with cystinuria [12,13].

This case series highlights the successful use of potassium sodium hydrogen citrate in dissolving kidney stones in patients with varying stone compositions and medical histories, underscoring its potential as an effective non-invasive treatment alternative that could improve patient outcomes and quality of life.

## Case presentation

This case report adheres to the CARE guidelines. The work described in this report was approved by the Institutional Review Board of King Abdullah International Medical Research Center, Riyadh, Saudi Arabia, IRB 0179/24. Written informed consent was obtained from the patients for publication.

Case 1

**Figure 1:**
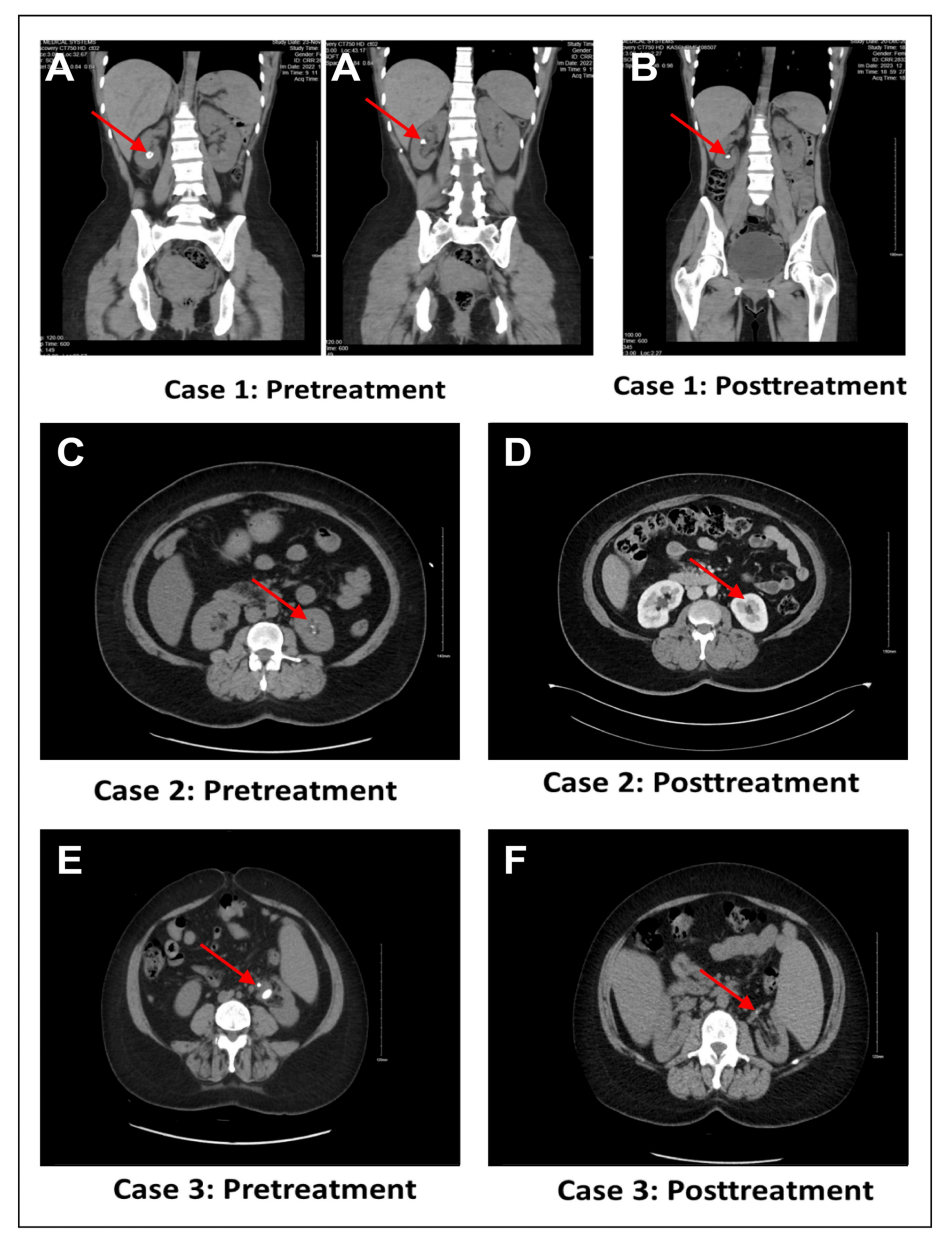
Computerized tomography images of case 1, case 2 and case 3 before and after treatment

A 29-year-old woman with a history of right breast mass (fibroadenoma), history of recurrent renal stones, and cystinuria had undergone multiple procedures, including PCNL and ureteroscopy. A computerized tomography (CT) scan of the kidneys, ureters, and bladder revealed multiple renal stones in the lower pole of the right kidney, the largest measuring 1.0 cm. Stone analysis showed calcium phosphate and cystine (Figures 1A, 1B). She was scheduled for elective ureteroscopy/ureterorenoscopy and started on potassium sodium hydrogen citrate 10 g thrice-daily (TID) for nine months preoperatively. After this, she underwent ureteroscopy which showed no residual stones, indicating complete dissolution.

Case 2

A 53-year-old woman with hypertension and a history of a large left renal stone measuring 2.8 cm had previously undergone PCNL. Follow-up CT scan revealed multiple remnant stones, the largest being 0.4 cm. Stone analysis showed calcium oxalate (Figures 1C, 1D). She was initiated on potassium sodium hydrogen citrate 2.4 g TID, and a subsequent CT scan conducted three months later revealed the absence of any residual stones.

Case 3

A 51-year-old woman with myelofibrosis transformed from polycythemia vera, recurrent urinary tract infections, and unilateral ureteropelvic junction obstruction (UPJO) had undergone pyeloplasty and stent insertion. Preoperative CT scans of the kidneys, ureters, and bladder showed a stone measuring 1.8 cm. No stone analysis was performed (Figures 1E, 1F). Postoperatively, the patient was treated with potassium sodium hydrogen citrate 2.4 g TID and was subsequently followed up in the clinic. A CT scan conducted three months later showed complete dissolution of the kidney stones.

Case 4

**Figure 2:**
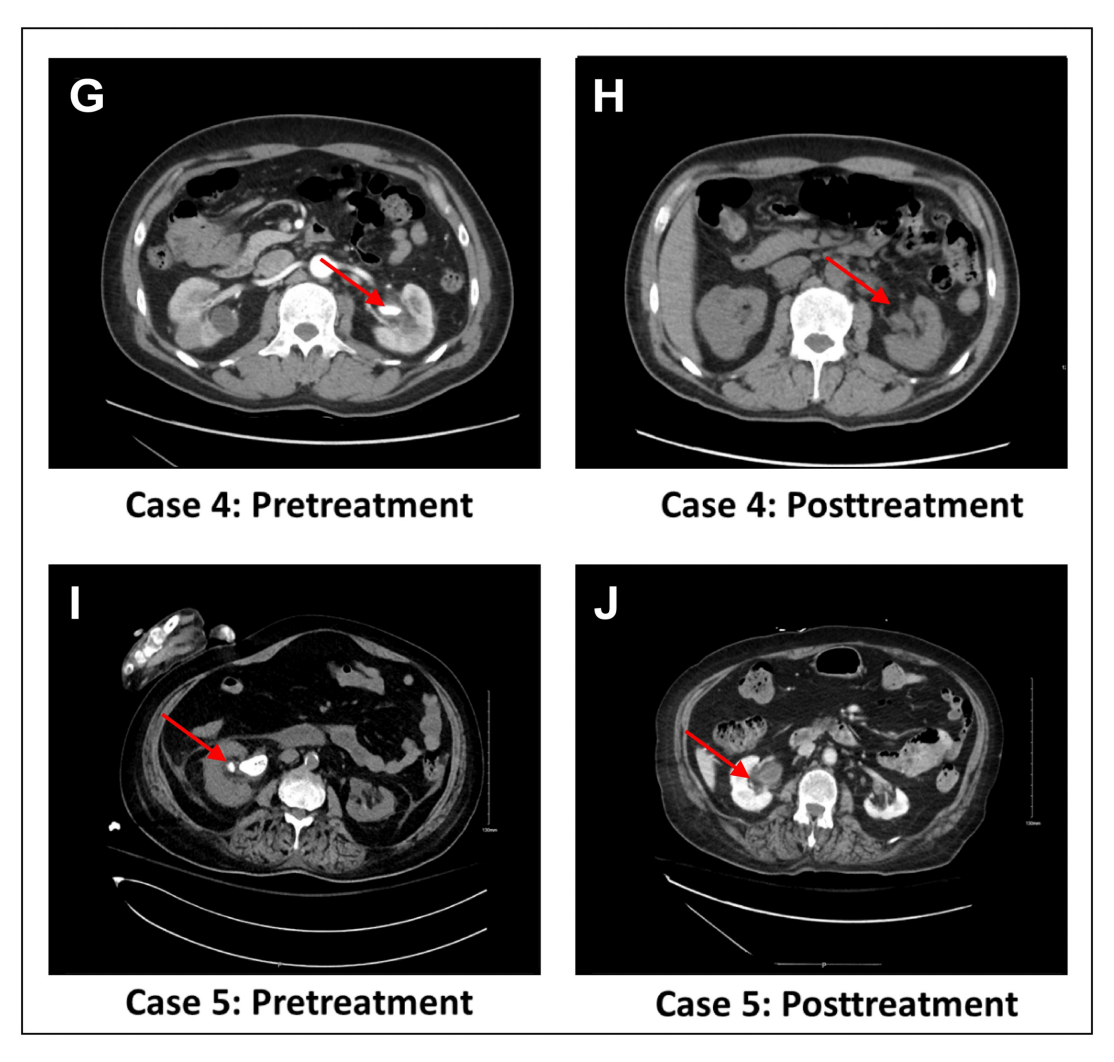
Computerized tomography images of case 4 and case 5 before and after treatment

A 48-year-old man with renal cell carcinoma, dilated cardiomyopathy with an automated implantable cardioverter defibrillator post-cardiac arrest, CKD, and chronic myelogenous leukemia was found to have an incidental left staghorn stone measuring 2 cm. No stone analysis was performed (Figures 2G, 2H). The patient was asymptomatic and not a candidate for surgery. He was treated with potassium sodium hydrogen citrate 2.4 g twice daily and followed up in the clinic. A CT scan six months later showed complete dissolution of the stone.

Case 5

A 72-year-old woman with multiple comorbidities, including diabetes mellitus, hypertension, dyslipidemia, heart failure, atrial fibrillation, and a history of stroke with poor functional status, was diagnosed with a left staghorn stone measuring 2.6 × 0.8 cm. No stone analysis was performed (Figures 2I, 2J). The patient experienced recurrent urinary tract infections and was deemed unfit for surgery. She was treated with potassium sodium hydrogen citrate 2.4 g TID. Three months later, a CT scan showed complete dissolution of the stones.

## Discussion

The management of kidney stones in the Middle East, particularly in Saudi Arabia, faces unique challenges due to the high prevalence and recurrence rates. This series of five cases underscores the efficacy of potassium sodium hydrogen citrate in dissolving kidney stones across various patient profiles and stone compositions. This non-invasive treatment approach offers a viable alternative for patients with complex medical histories, reducing the need for surgical interventions. Despite its proven effectiveness, the adoption of potassium sodium hydrogen citrate in Saudi Arabia remains limited, largely due to a prevailing preference for surgical procedures such as PCNL and ureteroscopy [14,15], favored for their immediate outcomes, especially for larger or more complex stones [14]. Moreover, the limited availability of robust local studies and inveterate clinical guidelines has contributed to a lack of awareness and confidence in nonsurgical interventions, like alkaline citrate, potentially hindering its broader adoption. A study also found that some urologists in Saudi Arabia have suboptimal knowledge of stone recurrence prevention programs, which affects their adherence to best practice guidelines for stone prevention [16]. Additional barriers, such as patient compliance and limited availability of medication, further hinder the adoption of potassium sodium hydrogen citrate in Saudi Arabia. The long-term use of this treatment may be challenging for patients with adherence issues, and its availability may be limited in certain regions, restricting access. Addressing these barriers is crucial to successfully integrating potassium sodium hydrogen citrate into routine clinical practice.

Moreover, patient procrastination, hesitancy to seek early consultation, and potentially long waiting times for appointments in the governmental healthcare system can delay the initiation of appropriate treatment, exacerbating the patient's condition. In this context, potassium sodium hydrogen citrate can help stabilize stones while patients await formal consultation or surgical procedures. This could reduce the size and hardness of stones, making them easier to manage surgically or even obviating the need for surgery altogether. Such an approach can also reduce the overall burden on the healthcare system by lowering the number of emergency cases requiring surgical intervention. However, caution is necessary when using potassium sodium hydrogen citrate in patients with CKD, as demonstrated in Case 4. The risk of hyperkalemia is a significant concern, as potassium citrate therapy can increase serum potassium levels, leading to serious complications, including cardiac arrhythmias. Careful monitoring of serum potassium levels is essential for patients with CKD undergoing this therapy, and kidney function should be assessed before initiating treatment. Additionally, calcium oxalate stone formers on potassium citrate therapy require monitoring, as elevated urine pH could contribute to the formation of calcium phosphate stones. The elevated urinary pH, combined with increased oxalate and phosphate levels, could lead to supersaturation of calcium phosphate. This suggests that alkaline citrate may not be beneficial for calcium phosphate stone formers, necessitating careful evaluation before use [17,18]. Regular monitoring of urine pH and calcium phosphate levels is crucial to minimizing the risk of stone formation associated with therapy.

The lack of long-term follow-up data on recurrence rates post-treatment is a significant limitation. Future studies should prioritize long-term monitoring to provide insights into the therapy’s preventive capabilities and refine treatment protocol. While the current case series demonstrates the complete dissolution of stones, the potential for recurrence over extended periods remains unaddressed. This highlights the need for studies focusing on long-term monitoring of patients treated with potassium sodium hydrogen citrate. Such research would provide valuable information on its preventive capabilities, helping to refine treatment protocols and optimize therapeutic strategies.

The successful outcomes observed in the case series illustrate the potential of alkaline citrate in managing kidney stones effectively. These cases demonstrate complete dissolution of staghorn stones, including those composed of calcium oxalate, and cystine, after a course of potassium sodium hydrogen citrate treatment. This suggests that alkaline citrate can be an effective nonsurgical option for a broad range of patients. Alkaline citrate works by increasing urinary citrate and pH levels, thereby reducing calcium crystallization and promoting the dissolution of stones. This approach not only prevents the formation of new stones but also aids in the dissolution of existing ones, making it a valuable tool in both the treatment and prevention of kidney stones.

The management of staghorn stones, which usually requires complex surgical procedures, showed promising results with potassium sodium hydrogen citrate in patients unsuitable for surgery. This suggests its potential to simplify treatment strategies for such cases. The promising results observed in these cases suggest that potassium sodium hydrogen citrate could play a pivotal role in transforming the management of kidney stones, particularly for patients who are not ideal candidates for surgical interventions. However, despite these promising outcomes, further research is needed to establish standardized treatment protocols and long-term outcomes. Future studies should include multicenter studies in Saudi Arabia and other regions with extended follow-up periods to evaluate recurrence rates and the overall cost-effectiveness of this approach. Such data would strengthen the evidence base for incorporating potassium sodium hydrogen citrate into routine clinical practice.

The therapeutic effect of alkaline citrate in preventing calcium oxalate crystal formation has been well documented [13,19-21]. For cystine stones, which are relatively insoluble at physiological urine pH levels and have high recurrence rates, urinary alkalinization with potassium citrate (pH >7.5) helps prevent their formation [22]. Citrate therapy is generally well-tolerated, with the most common side effects being gastrointestinal discomfort (nausea and bloating). However, potassium sodium hydrogen citrate should not be used in cases of impaired renal excretory function, metabolic alkalosis, hyperkalemia, adynamia episodica hereditaria, chronic urinary tract infections with urea-splitting bacteria (due to the danger of formation of struvite stones), and low sodium diet. Before starting treatment, all circumstances or diseases that may favor urinary stones and for which well-targeted therapy is available (such as adenoma of parathyroid glands or malignancy associated with uric acid stones) should be ruled out. Prior to the first dose, serum electrolytes should be determined, and renal function should be monitored. Potassium sodium hydrogen citrate should also be used with caution in patients with severely impaired liver function. These comprehensive considerations underscore the importance of tailored patient assessments before initiating potassium sodium hydrogen citrate therapy to ensure both safety and efficacy in the management of kidney stones.

## Conclusions

The presented cases highlight the significant potential of potassium sodium hydrogen citrate in the management of kidney stones. Despite the prevalent preference for surgical interventions among urologists in Saudi Arabia, chemical management demonstrates an effective non-invasive alternative that can manage stones of various compositions, prevent recurrence, and serve as a valuable interim solution for patients awaiting surgical treatment or for those deemed unfit for surgery. Incorporating alkaline citrate into standard treatment protocols could enhance patient outcomes, reduce healthcare costs, and address challenges related to timely healthcare access.

To fully realize its potential, specific next steps should include conducting multicenter clinical studies, developing evidence-based guidelines for prescribing potassium sodium hydrogen citrate, and exploring its cost-effectiveness in diverse healthcare settings. These efforts will help bridge the gap between clinical evidence and routine clinical practice, ensuring broader adoption and improved patient outcomes.
